# Efficacy of Chinese Herbal Medicine in the Treatment of Moderate-Severe Painful Diabetic Peripheral Neuropathy: A Retrospective Study

**DOI:** 10.1155/2019/4035861

**Published:** 2019-12-23

**Authors:** Yujiao Zheng, Fan Yang, Lin Han, Xiaowen Gou, Fengmei Lian, Wenke Liu, Linhua Zhao, Bing Pang, Xiyan Zhao, Xiaolin Tong

**Affiliations:** ^1^Department of Endocrinology, Guang'anmen Hospital, China Academy of Chinese Medical Sciences, China; ^2^Graduate School, Beijing University of Chinese Medicine, China; ^3^Rehabilitation Hospital Affiliated to National Research Center for Rehabilitation Technical Aids, China

## Abstract

Painful diabetic peripheral neuropathy (pDPN) is a debilitating complication of diabetes. The aim of this retrospective study was to investigate the effectiveness of a Chinese herbal medicine regimen—the modified Huangqi Guizhi Wuwu Decoction (HGWD)—in the treatment of moderate-severe pDPN. The primary objective was to estimate the improvement in neuropathic pain severity. The secondary objective was to assess the response of common symptoms to the treatment. The change in patients' blood glucose level during the whole treatment was also evaluated. By searching through our medical records of all the diabetic patients from January 2006 to January 2012, we identified and enrolled 30 moderate and severe pDPN patients in the study, for whom the treatment of neuropathic pain by regular pharmacotherapies had failed. The modified HGWD treatment was administered orally twice a day for 6 months. The numerical rating scale (NRS) level at month 6 was 2.57 ± 2.30, significantly improved compared with the baseline level of 6.03 ± 1.83 (*P* < 0.05). The amelioration of 3 common symptoms, namely, limb pain, limb numbness, and insomnia, was evident, and the major response of common symptoms at month 6 including limb pain, insomnia, and limb coldness was significantly increased compared with the results at month 3 (*P* < 0.05). Moreover, 2-hour postprandial blood glucose (2hPG) level decreased from 10.77 ± 1.29 mmol/L at baseline to 9.66 ± 0.60 mmol/L at month 6 (*P* < 0.05). No serious adverse events occurred throughout the treatment period. The modified HGWD was effective in the treatment of moderate and severe pDPN and can thus be offered as a new alternative treatment option for pDPN patients who failed to respond to regular pharmaceutical therapies.

## 1. Introduction

Diabetic peripheral neuropathy (DPN), one of the most common complications of diabetes mellitus, is defined as the presentation of symptoms and/or signs of peripheral nerve dysfunction in patients with diabetes after the exclusion of other causes [1]. It has been estimated that approximately 50% of type 1 and type 2 diabetic patients are affected by DPN [2]. The typical manifestations of DPN include various neuropathic symptoms, including burning, stabbing, or shooting pain, and numbness in the extremities, which is especially intense at night. Among these symptoms, neuropathic pain is the most significant and the most difficult to tolerate [3]. It is estimated that at least 3%~25% of the diabetic population suffers from neuropathic pain, also called painful DPN (pDPN) [4]. Compared with painless DPN, pDPN has a more significant debilitating impact on physical and mental performance [5]. A study from the U.K. showed that the prevalence of pDPN was 60% among patients affected by severe DPN [6]. As one of the severest and most disabling complications of diabetes mellitus, pDPN leads to impaired quality of life and increased mortality, resulting in a significant healthcare burden [7]. Pharmaceutical interventions are the primary therapeutic options for the treatment of pDPN and are mainly focused on treating the symptoms [8, 9]. Effective and widely prescribed pharmacotherapies include anticonvulsants, antidepressants, opiate-receptor agonists, and topical agents [10]. Among them, pregabalin and duloxetine are the first-line therapies for pDPN and have been approved by the US Food and Drug Administration [11, 12]. However, pDPN patients are often subject to polypharmacy and comorbidities related to hyperglycemia and other microvascular and macrovascular complications, increasing the risk of drug-drug and drug-condition interactions [13].

Traditional Chinese medicine (TCM), which has a thousand years of history in China and other Asian countries, is one of the major systems of complementary and alternative medicine. As the predominant branch of TCM, Chinese herbal medicine (CHM) is currently widely applied in the treatment of many diseases. The flexible modifications of herbal medicines, with various functions performed by each prescription, and the essential holistic theory of TCM allow CHM to reduce drug-drug and drug-condition interactions [14]. Recently, CHM has been playing a progressively more important role in the treatment of diseases with complex pathogenesis and symptoms, including DPN. Both clinical trials and animal tests have proved the efficacy of CHM in the treatment of DPN, but more studies are needed to accumulate further evidence [15–17].

Huangqi Guizhi Wuwu Decoction (HGWD), which was first recorded in a famous Chinese medical classic named *Treatise on Cold-induced Febride Diseases* in the Han Dynasty (3^rd^ century AD). As a commonly used CHM formula able to stimulate blood circulation and enhance body motility, HGWD has been widely used in DPN for decades [18]. We have reported previously that HGWD in combination with aconitum exerted promising effects in attenuating neuropathic pain in a severe pDPN patient [19]. To further examine the efficacy of HGWD in the treatment of pDPN, we present here a retrospective case series of 30 moderate or severe pDPN patients treated with modified HGWD.

## 2. Materials and Methods

### 2.1. Data Source

This study was a retrospective case series. Data for the study were obtained from the medical records of all diabetic patients who consulted our outpatient clinic of the Endocrinology Department in the Guang'anmen Hospital between January 2006 and January 2012. The study was approved by the Ethics Committee of Guang'anmen Hospital (Identification No. 20190614).

### 2.2. Patient Selection

Diabetic patients with moderate or severe neuropathic pain were enrolled in the study based on the following inclusion criteria: (1) pDPN diagnosed before patients were treated with modified HGWD, as determined by the patients' previous medical records; (2) failure of the treatment of neuropathic pain by regular pharmacotherapies such as pregabalin, duloxetine, or other therapeutic interventions; (3) discontinuation of all these interventions before treatment with modified HGWD in our outpatient clinic; (4) moderate or severe neuropathic pain present when they first received our modified HGWD treatment, with pain score at least 4 according to the numeric rating scale (NRS) ranging from 0 to 10, where 0 = no pain and 10 = worst possible pain [20]; (5) modified HGWD treatment administered for at least 6 months and the frequency of follow-up visits which was at least once every 2 months; and (6) no other therapeutic interventions for neuropathic pain administered during the whole treatment period. Patients were excluded if they met one or more of the following conditions: (1) other types of neuropathic pain diagnosed or concomitant, based on the patients' medical histories or laboratory tests, and (2) modified HGWD not taken continuously for the entire duration of the treatment.

### 2.3. Treatment

In this study, modified HGWD was prescribed for the treatment of neuropathic pain in pDPN patients. Any previous regime of oral hypoglycemic drugs or insulin for the control of blood glucose was maintained. The formula of modified HGWD was established based on the TCM theory of nourishing *Qi* and invigorating blood circulation. The basic formula consisted of 6 herbs: Astragali Radix (Huangqi in Chinese), Cinnamomi Ramulus (Guizhi in Chinese), Spatholobi Caulis (Jixueteng in Chinese), Paeoniae Radix Alba (Baishao in Chinese), Polygoni Multiflori Caulis (Shouwuteng or Yejiaoteng in Chinese), and Glycyrrhizae Radix (Gancao in Chinese) (Table 1). If the patients felt intolerable pain, the herbs Notoginseng Radix (Sanqi in Chinese), Aconitum Radix (Wutou in Chinese), Olibanum (Ruxiang in Chinese), and Myrrha (Moyao in Chinese) were added. If the blood glucose of the patients was too high, the herbs Coptidis Rhizoma (Huanglian in Chinese) and Anemarrhenae Rhizoma (Zhimu in Chinese) were added. Moreover, the herbs Hirudo (Shuizhi in Chinese) and Rheum Officinale (Dahuang in Chinese) were added in case of abnormal urinary protein content. Every prescription was decided by Professor Tong Xiaolin, which is based on the combination of the individual's syndromes and symptoms in TCM theory. According to Professor Tong's experience on the treatment of DPN, the common dose of the basic formula is Astragali Radix 30 g, Cinnamomi Ramulus 15 g, Spatholobi Caulis 30 g, Paeoniae Radix Alba 15 g, Polygoni Multiflori Caulis 30 g, and Glycyrrhizae Radix 15 g. However, due to the characteristics of individualized prescription of TCM clinical diagnosis and treatment in outpatients, the dosage of every herb in each individual prescription was not totally fixed.

On the basis of the patients' preference, modified HGWD was administered in the form of decoction due to their trust in a traditional decoction method. The Chinese medicine material crude slices used for decoction were supplied and quality controlled by Kangmei Pharmaceutical Co., Ltd. (Beijing, China), which were conformed to meet the standard of China Pharmacopoeia (2015 edition) [21]. All herbal material crude slices were purchased in the pharmacy of Guang'anmen Hospital (Beijing, China) by the patients themselves. The preparation of the modified HGWD decoction was based on a standard progress. The herbal material crude slices were weighted and soaked into 10 times of the herbal materials weight of cold water for 1 hour. Then, the herbs were boiled for 50 minutes, filtrated, and concentrated to a final volume of 300 mL decoction. The decoction (150 mL) was taken orally twice a day. The chemical composition of the basic formula of modified HGWD decoction was analyzed and profiled using a high-performance liquid chromatography (HPLC) system (model 2695, Waters, Milford, Ireland), equipped with a vacuum degasser, a quaternary pump, an autosampler, and a photodiode array (PDA) detector. Nine chemical components, namely, catechin, epicatechin, paeoniflorin, calycosin, liquiritin, stilbeneglycoside, cinnamaldehyde, formononetin, and glycyrrhizin, were used in the qualitative analysis, and a HPLC fingerprint of the decoction was obtained ([Supplementary-material supplementary-material-1]).

### 2.4. Study Measures

Data from the patients' medical records were manually collected and examined manually by two researchers from our outpatient clinic. The demographic and clinical characteristics of the pDPN patients were recorded at baseline, including age, sex, body mass index (BMI), and diabetes history (type, duration, and comorbidities). The effectiveness of the modified HGWD treatment was recorded and evaluated at 3 and 6 months compared with baseline, based on the primary and secondary objectives. Additional telephone interviews were conducted when needed to supplement the data obtained from the medical records.

The primary objective was to estimate the improvement in neuropathic pain severity in these pDPN patients. Neuropathic pain severity was measured by the score of the numeric rating scale (NRS) as reported in the medical records.

The secondary objective was to assess the response of common symptoms, where “major response” referred to improvement by 50% or more, “minor response” referred to improvement less than 50%, and “no response” referred to no improvement or worsening. The response rate was defined as the sum of the major response rate and minor response rate. The reproducibility of the rating method was demonstrated by a separate, blinded grading by two researchers on the patients' medical records.

The change in patients' blood glucose during the whole treatment was also evaluated, including glycated hemoglobin level (HbA1c), fasting blood glucose (FBG), and 2-hour postprandial blood glucose (2hPG). The measurements were carried out in the laboratory of Guang'anmen Hospital.

### 2.5. Statistical Analysis

All data were analyzed using the SPSS 20.0 software (SPSS Inc., Chicago, IL, United States). Regarding demographic and clinical characteristics at baseline, quantitative variables were reported as the mean ± standard deviation (SD) and qualitative variables as frequencies and percentages. The severity rate of neuropathic pain measured by the NRS score was presented as the mean ± SD. Common symptoms were defined as the 10 symptoms most frequently occurring in our cohort at baseline, month 3, and month 6. HbA1c, FBG, and 2hPG levels were reported as the mean ± SD. Since some patients neglected to undergo laboratory examinations at the time of return visits, missing data were processed by deleting the records with too many missing variables, then interpolating with the mean values to replace missing values in the remaining records. One-way ANOVA was used to compare NRS scores among the groups, and one-way ANOVA followed by a LSD (Least Significant Difference) post hoc test was used to compare HbA1c, FBG, and 2hPG levels among different times. A chi-square test was used to compare the response rate of common symptoms between months 3 and 6. *P* values < 0.05 were considered statistically significant.

## 3. Results

### 3.1. Demographic and Patient Characteristics

By searching our medical records of all the diabetic patients from January 2006 to January 2012, 30 patients (15 males and 15 females) were eligible according the inclusion and exclusion criteria and were enrolled in this study. The mean age of the included patients was 59.00 ± 12.28 years (range: 20 to 75 years). Twenty-nine patients (96.67%) had been diagnosed with type 2 diabetes and only one (3.33%) with type 1 diabetes. The mean duration of diabetes at the time of diagnosis was 10.32 ± 7.50 years (range: 0.5 to 40 years), and the mean BMI was 23.60 ± 3.39 kg/m^2^ (range: 15.6 to 31.2 kg/m^2^, Table 2). Among the 30 patients, the most common comorbidities were hypertension (53%), hyperlipidemia (27%), and diabetic retinopathy (27%, Table 3).

### 3.2. Change in Neuropathic Pain Severity

**Figure 1:**
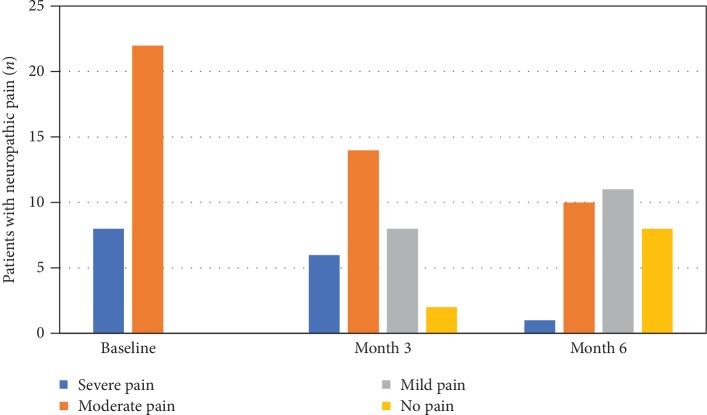
Change in the frequency of neuropathic pain. After 3 months of modified HGWD, 2 patients recovered from moderate neuropathic pain, 8 patients turned from moderate to mild neuropathic pain, and 2 turned from severe to moderate neuropathic pain, while 18 patients (12 with moderate and 6 with severe pain) remained unchanged. After 6 months, 8 patients recovered from moderate neuropathic pain, 10 patients turned from moderate to mild neuropathic pain, 1 patient turned from severe to mild neuropathic pain, and 6 patients with severe pain turned to moderate pain, while only 5 patients remained unchanged.

Moderate neuropathic pain was defined as 4 to 6 NRS points and severe pain as 7 to 10 points. Among the 30 pDPN patients, 22 had moderate neuropathic pain (73.33%) and 8 were evaluated to have severe neuropathic pain (26.67%) at baseline. The mean NRS score was 6.03 ± 1.83. All patients had received regular pharmacotherapies for treating neuropathic pain without satisfactory results before visiting our outpatient clinic. After being treated with modified HGWD for 3 months, 2 patients recovered from moderate neuropathic pain and did not require further medical attention, 8 patients turned from moderate to mild neuropathic pain, and 2 turned from severe to moderate neuropathic pain, while 18 patients (12 with moderate and 6 with severe pain) remained unchanged. The mean NRS level was 4.20 ± 2.41, and the difference with baseline was significant (*P* < 0.05). After 6 months of modified HGWD treatment, compared with baseline, 8 patients recovered from moderate neuropathic pain, 10 patients turned from moderate to mild neuropathic pain, 1 patient turned from severe to mild neuropathic pain, and 6 patients with severe pain turned to moderate pain, while only 5 patients remained unchanged. The mean NRS level at month 6 was 2.57 ± 2.30, and the improvement was statistically significant compared with both baseline (*P* < 0.001) and month 3 (*P* < 0.05, Table 4, Figure 1).

### 3.3. Change in Common Symptoms

Symptom frequency was calculated based on the medical records of the 30 patients at baseline, month 3, and month 6, and the top 10 symptoms were identified as common symptoms. These were limb pain, limb numbness, insomnia, limb coldness, difficulty walking, blurred vision, constipation, fatigue, limb heaviness, and chills (Table 5). Compared with baseline, the improvement of limb pain, limb numbness, and insomnia were the most evident. Nineteen patients showed major response, and 10 showed minor response in limb pain at month 6, implying that 29 patients in total (96.67%) benefited from the improvement of limb pain after our modified HGWD treatment. With respect to limb numbness, 14 patients showed major response and 12 showed minor response at month 6, so 26 out of the 30 cases (86.67%) showed beneficial response. Moreover, 18 patients showed major response and 5 showed minor response regarding insomnia at month 6, with a total of 23 patients (76.67%) showing beneficial response. The major response rate and response rate of common symptoms at months 3 and 6 were also compared. The result showed that the major responses of limb pain and insomnia at month 6 were remarkably increased compared with the results at month 3 (*P* < 0.01), and the major response of limb coldness at month 6 was also significantly increased after 3 months (*P* < 0.05). However, the response rate of common symptoms, which was the sum of the major response rate and minor response rate between months 3 and 6 showed no obvious difference (Table 6).

### 3.4. Change in Blood Glucose Level

The change in the blood glucose level was analyzed in terms of HbA1c, FBG, and 2hPG measurements at baseline, month 3, and month 6. The HbA1c concentration was 7.12 ± 0.82% at baseline and decreased to 6.80 ± 0.78% by 6 months, but there were no significant differences comparing baseline, month 3, and month 6 (*P* > 0.05). The mean FBG level was 8.37 ± 2.65 mmol/L at baseline and decreased to 7.01 ± 1.14 mmol/L at month 6, but there were no significant differences comparing baseline, month 3, and month 6 (*P* > 0.05). The 2hPG level was 10.77 ± 1.29 mmol/L at baseline and decreased to 9.66 ± 0.60 mmol/L after 6 months of treatment; the difference with both baseline and month 3 was statistically significant (*P* < 0.05) (Table 7).

During the 6 months of treatment, there was no report of any serious adverse events, and none of the patients progressed to diabetic foot ulceration or amputation.

## 4. Discussion

In the present retrospective case series study, we investigated the efficacy of modified HGWD treatment on pDPN by analyzing and evaluating the medical records of 30 pDPN patients with moderate or severe neuropathic pain. The study applied NRS to measure the neuropathic pain level of the pDPN patients. NRS is one of the widely accepted pain rating scales which are commonly used in judging neuropathic pain in DPN. Compared with other scales, NRS has greater sensitivity and is the choice of most people in clinical practice [22]. Our results showed that NRS levels declined significantly after 3 and 6 months of treatment, demonstrating that the modified HGWD treatment had a clinical effect in ameliorating the neuropathic pain of pDPN patients. Besides, the modified HGWD also improved other clinical symptoms, such as limb pain, numbness, and insomnia, and the major responses of limb pain, insomnia, and limb coldness at month 6 were significantly increased in comparison with the results of month 3, indicating that the improvement of these symptoms may have a time-dependent trend. Compared with conventional Western pharmaceutical therapies, HGWD provided simultaneous treatment on various symptoms, in agreement with the holistic philosophy and multitarget characteristics of TCM theory [23]. The results of the study also showed improvement of 2hPG levels during the whole treatment, while the effects on HbA1c and FBG reduction were not significant. However, it is not clear whether the hypoglycemic effect was obtained by the hypoglycemic efficacy of modified HGWD or good glycemic control. Furthermore, no adverse events were detected throughout the whole treatment, proving the safety of our CHM application. With respect to the deterioration of pDPN, no diabetic foot ulcerations or amputations were recorded in these 30 pDPN patients after 6 months of treatment, suggesting that the modified HGWD contributes to preserve the quality of life.

To our knowledge, the present study is one of the very few clinical studies showing a promising effect on pDPN patients with moderate and severe peripheral neuropathic pain by the oral administration of complementary and alternative medicine, with the added advantage of providing a comprehensive, multitarget effect on various clinical manifestations of pDPN. Other complementary and alternative medicine practices, such as acupuncture (including manual acupuncture and electroacupuncture) and acupoint injection, were also shown to lead to pain improvement in DPN [24–26]. Topical capsaicin, turpentine oil, and Citrullus colocynthis (bitter apple) extract oil application over the pain sites were also reported to be effective for pDPN [27–29]. Moreover, aromatherapy massage has been described as a feasible complementary and alternative medicine approach to relieve neuropathic pain in pDPN patients [30]. However, the current clinical studies about the treatment of pDPN by complementary and alternative medicine are mostly related to nonoral, topical therapies. Besides, these studies mainly focus on DPN patients with mild or moderate neuropathic pain. Therefore, our study provides a new approach for pDPN patients experiencing moderate to severe neuropathic pain.

DPN usually occurs in mid- or late-stage diabetes, and TCM theory considers *Qi* deficiency to appear at this time. According to TCM, the movement of *Qi* is a significant factor in maintaining health during the whole lifespan, as it promotes blood circulation [31]. During the progress of pDPN, *Qi* is consumed and gradually becomes deficient, leading to blood stasis. Many herbs and minerals were confirmed to have the function of invigorating *Qi* and blood movement. However, TCM practitioners often use a combination of multiple herbs or minerals to compose CHM formulas based on TCM principles, instead of a single herb or mineral, an approach that focuses on enhancing clinical efficacy by targeting complex pathogenetic mechanisms. In Tong's outpatient clinic, he applied the principle of nourishing *Qi* and promoting blood circulation to treat pDPN patients and chose the HGWD regimen including some herbs with the specific function of relieving neuropathic pain [19, 32].

HGWD, a classic CHM formula, has been used by TCM practitioners for the clinical treatment of DPN for a long time. There are evidences that HGWD improves DPN clinical symptoms and ameliorates nerve conduction velocity while decreasing the plasma viscosity of DPN patients [33, 34]. A previous study has reported that the mechanism of HGWD on neurological function improvement in DPN is associated with oxidative stress inhabitation through nuclear factor-E2-related factor 2 (Nrf2) and B-cell CLL/lymphoma 2 (Bcl2) pathway [35]. In this present study, Spatholobi Caulis, Polygoni Multiflori Caulis, and Glycyrrhizae Radix were added in HGWD to improve the clinical efficacy in DPN with moderate or severe neuropathic pain. With the development of pharmacology and basic researches on CHM, the therapeutic mechanism and pharmacological action of herbs in the modified HGWD are being explored. As we mentioned above, Astragali Radix is one of the main components of modified HGWD. In TCM theory, Astragali Radix has the effect of tonifying *Qi*, and *Qi* deficiency is considered a primary factor for the occurrence of neuropathic pain [36]. Pharmacology studies found that one of the main active components of Astragali Radix, Astragaloside IV (AS-IV), has neuroprotective effects and promotes neovascularization and is also indicated for the prevention of diabetes and its complications because of its decrease in lowing blood glucose and HbA1C levels and increase in insulin levels. The mechanisms involve increasing glutathione peroxidase activity in nerves, inhibiting aldose reductase activation, and lowering advanced glycation end products in both nerves and erythrocytes [37, 38]. Cinnamomi Ramulus is often combined with Astragali Radix in TCM applications for the treatment of *Qi* deficiency diseases. It has been proved that the active component of Cinnamomi Ramulus, cinnamomi acid, exerts an analgesic action against neuropathic pain by inhibiting spinal pain transmission, and the mechanism may be related to its agonist action on transient receptor potential ankyrin 1 (TRPA1) [39, 40]. Spatholobi Caulis activates blood flow and promotes blood circulation, which is beneficial for blood stasis. And blood stasis, as discussed above, is considered by TCM a significant pathogenetic mechanism of pDPN. Recently, it demonstrated that the extracts of Spatholobi Caulis have neuroprotective effects and inhibit the effects of diabetes-induced glucotoxicity for its improvement in apoptotic phenotypes and oxidative stress [41, 42]. Paeoniae Radix Alba is known for its analgesic activity and has been widely used to treat pain-related diseases with Glycyrrhizae Radix; it has been recently explored that the combination of two herbs possesses an obvious analgesic and anti-inflammatory effect and the potential molecular mechanism might be due to the increasing expression and function of transient receptor potential ion channel protein vanilloid receptor 1 (TRPV1) channel [43]. Another study also elucidated that the mechanism of Paeoniae Radix Alba's hypoglycemic and neuroprotective effects in the treatment of DPN might be associated with the Nrf2/antioxidant response element (ARE) pathway and Bcl-2 related apoptotic pathway [44]. Polygoni Multiflori Caulis is a common herb often used for insomnia, which we added to ameliorate the clinical manifestations of sleeplessness resulting from neuropathic pain [45]. Studies have shown that the extracts of Polygoni Multiflori Caulis have a neuromodulation effect [46]. The extracts of Polygoni Multiflori Caulis also have hypoglycemic and hypolipidemic effects; the mechanisms are built on the inhibition of *α*-glucosidase and fatty acid synthase (FAS) activity [47, 48].

The present study has some limitations. First, due to its retrospective nature and its reliance on past medical records, some important data were inevitably missed or difficult to obtain. Second, the study did not have a control group because it is rather difficult for us to enroll moderate to severe pDPN patients who have not taken Chinese medicine in the clinic, so we could not present a comparison of pDPN patients with and without modified HGWD treatment. Moreover, long-term outcomes and quality of life were not observed, because the follow-up was limited. Despite these limitations, we believe the present study is of value since it is the first clinical study to explore the effect of oral administration of complementary and alternative medicine on pDPN patients with moderate to severe neuropathic pain. The study can lay a foundation for later researches of complementary and alternative medicine in the treatment of DPN patients who suffers intolerable neuropathic pain.

In order to obtain evidence of higher quality on the efficacy of modified HGWD in the treatment of pDPN, our team has conducted a prospective, double-blinded, randomized controlled trial to further explore the curative effects, safety, and feasibility.

## 5. Conclusion

In summary, modified HGWD appeared to be effective and safe in the treatment of DPN with moderate or severe neuropathic pain, thus presenting a new alternative option for pDPN patients who failed to respond to regular pharmaceutical therapies. During our 6 months of study, pain relief and improvement of common symptoms were clearly shown. However, further studies like randomized clinical trials are inevitably needed to obtain higher quality evidence on the effects of modified HGWD on DPN, particularly in the long term.

## Figures and Tables

**Table 1:** Composition of modified HGWD.

Latin name	English name	Part used	Type of product	Common dose (g)
*Astragalus membranaceus* (Fisch.) Bge.	Astragali Radix	Root	Raw (dry)	30
*Cinnamomum cassia* Presl	Cinnamomi Ramulus	Twig	Raw (dry)	15
*Spatholobus suberectus* Dunn	Spatholobi Caulis	Cane	Raw (dry)	30
*Paeonia lactiflora* Pall.	Paeoniae Radix Alba	Root	Raw (dry)	15
*Polygonum multiflorum* Thunb.	Polygoni Multiflori Caulis	Cane	Raw (dry)	30
*Glycyrrhiza uralensis* Fisch.	Glycyrrhizae Radix	Root and cane	Fried (dry)	15

**Table 2:** Demographic and patient characteristics at baseline.

Clinical characteristic	
Age (yr)	59.0 ± 12.28^a^
Sex (F/M)	15 (50%)/15 (50%)
BMI (kg/m^2^)	23.60 ± 3.39^a^
Diabetes mellitus	
Type 1	1 (3%)
Type 2	29 (97%)
Duration of diabetes (yr)	10.32 ± 7.50^a^
Degree of pain	
Moderate	22 (73%)
Severe	8 (27%)

^a^Continuous variables reported as the mean ± SD.

**Table 3:** Comorbidities of patients.

Comorbidity	Frequency	Percentage (%)
Hypertension	16	53%
Hyperlipidemia	8	27%
Diabetic retinopathy	8	27%
Diabetic nephropathy	5	17%
Cataract	3	10%
Stroke	3	10%
Coronary heart disease	2	7%
Chronic gastritis	2	7%
Thyroid nodule	2	7%
Virus hepatitis	1	3%
Alcoholic hepatitis	1	3%

**Table 4:** Change in neuropathic pain severity.

	Baseline	Month 3	Month 6
NRS	6.03 ± 1.83	4.20 ± 2.41*^Δ^*	2.57 ± 2.30^*ΔΔ*○^

*P* values are calculated for the comparisons of month 3 with baseline and of month 6 with month 3 and with baseline. *^Δ^p* < 0.05 (compared with baseline); *^ΔΔ^P* < 0.001 (compared with baseline); ^○^*P* < 0.05 (compared with month 3).

**Table 5:** Frequency of common symptoms.

Main symptoms	Total frequency	Baseline	Month 3	Month 6
Limb pain	80	30	28	22
Limb numbness	68	28	24	16
Insomnia	48	24	15	9
Limb coldness	43	17	15	11
Difficulty walking	24	9	9	6
Blurred vision	19	7	7	5
Constipation	18	8	6	4
Fatigue	18	10	6	2
Limb heaviness	17	7	6	4
Chills	14	5	5	4

**Table 6:** Outcome of common symptoms.

Main symptoms	Intervention time (months)	Major response (*n*)	Minor response (*n*)	No response (*n*)	Major response rate (%)	Response rate (%)	*P* value 1	*P* value 2
Limb pain	3 6	8 19	16 10	6 1	26.67 63.33	80.00 96.67	0.004^∗∗^	0.103

Limb numbness	3 6	8 14	15 12	5 2	28.57 50.00	82.14 92.86	0.101	0.422

Insomnia	3 6	9 18	10 5	5 1	37.50 75.00	79.17 95.83	0.005^∗∗^	0.188

Limb coldness	3 6	3 9	9 6	5 2	17.65 52.94	70.59 88.24	0.031^∗^	0.398

Fatigue	3 6	5 8	3 1	2 1	50.00 80.00	80.00 90.00	0.350	1.000

Difficulty walking	3 6	1 5	5 4	3 0	11.11 55.56	66.67 100.00	0.131	0.206

Constipation	3 6	2 5	3 1	3 2	25.00 62.50	62.50 75.00	0.315	1.000

Blurred vision	3 6	2 3	2 3	3 1	28.57 42.86	57.14 85.71	1.000	0.559

Limb heaviness	3 6	2 5	4 2	1 0	28.57 71.43	85.71 100.00	0.286	1.000

Chills	3 6	1 2	2 2	2 1	20.00 40.00	60.00 83.33	1.000	1.000

*P* values were calculated for the comparison of major response rate and response rate between the results in month 3 and month 6. ^∗^*P* < 0.05; ^∗∗^*P* < 0.01.

**Table 7:** Change in blood glucose level.

	Baseline	Month 3	Month 6
HbA1c	7.12 ± 0.82	7.05 ± 0.88	6.80 ± 0.78
FBG	8.37 ± 2.65	8.26 ± 2.79	7.02 ± 1.14
2hPG	10.77 ± 1.29	12.48 ± 3.38	9.66 ± 0.60^∗^^#^

*P* values were calculated for the comparisons of month 3 with baseline and of month 6 with month 3 and with baseline. ^∗^*P* < 0.05 (compared with baseline); ^#^*P* < 0.05 (compared with month 3).

## Data Availability

The data used to support this study are available from the corresponding author upon request.

## Supplementary Materials

Supplementary Materials The protocol for chemical analysis of modified HWGD and the HPLC fingerprint were are available in the Supplementary Material.
