# Supplementary material for: Efficacy of Chinese Herbal Medicine in the Treatment of Moderate-Severe Painful Diabetic Peripheral Neuropathy: A Retrospective Study
Source: J Diabetes Res. 2019 Dec 23;2019:4035861. doi: 10.1155/2019/4035861 (PMC6948321; doi:10.1155/2019/4035861)
Supplement: Supplementary Materials — The protocol for chemical analysis of modified HWGD and the HPLC fingerprint were are available in the Supplementary Material. [file 4035861.f1.pdf]

Fig S1 Protocol for chemical analysis and the HPLC fingerprint of modified HGWD

(I) HPLC of nine standards

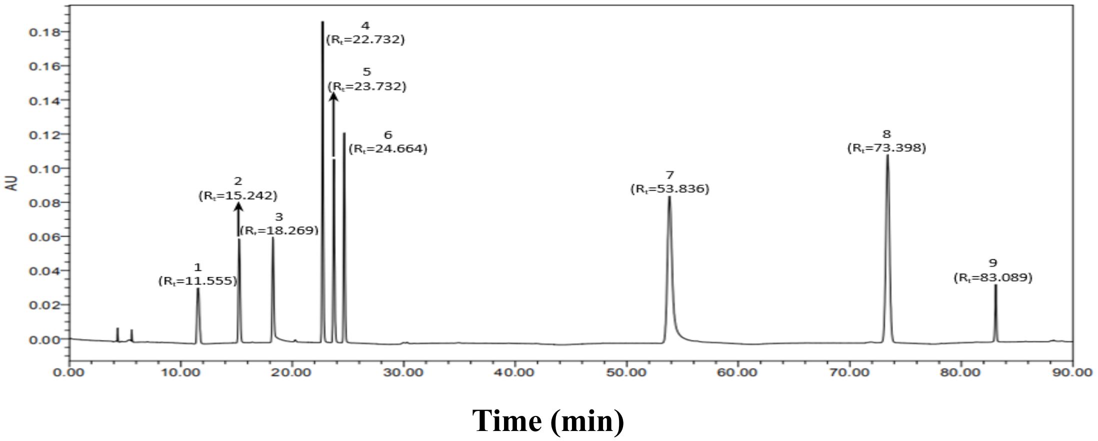

(II) HPLC of modified HGWD

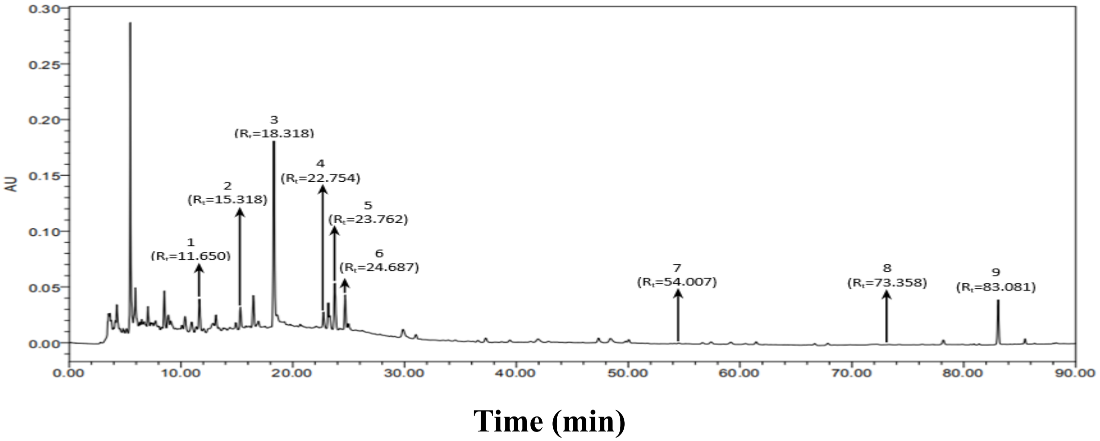

(III) Chemical structures of assayed compounds

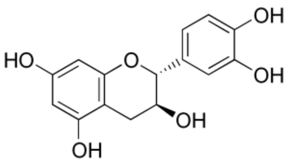

1. Catechin

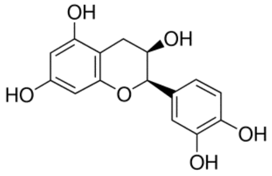

2. Epicatechin

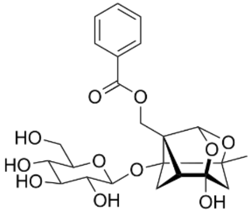

3. Paeoniflorin

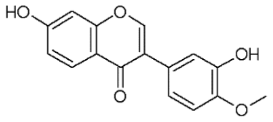

4. Calycosin

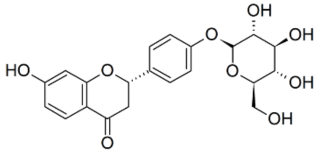

5. Liquiritin

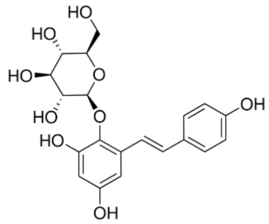

6. Stilbeneglycoside

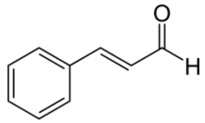

7. Cinnamaldehyde

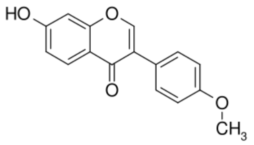

8. Formononetin

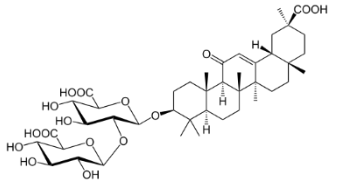

9. Glycyrrhizin

Fig S1: The chemical composition of modified Huangqi Guizhi Wuwu Decoction (HGWD) using high-performance liquid chromatography (HPLC) analysis. Modified HGWD was separated on a Luna®Omega Polar C18 (250 mm × 4.6 mm, 3 µm) column. The mobile phase was composed of acetonitrile (A) and 0.1% phosphoric acid (v/v) (B) using the following gradient program: 0–5 min, 14% A; 5–20 min, 14–23% A; 20–30 min, 23–24% A; 30–40 min, 24–27% A; 40–45 min, 27–30% A; 45–70 min, 30–35% A; 70–80 min, 35–45% A; 80–90 min, 45–60% A; the injection volume was 20 µL; the flow rate was 0.6 mL/min; the column

temperature was maintained at 35 °C; the effluent was monitored at 240 nm. Data acquisition and processing were performed by Empower software. The result compound peaks in the chromatograms include the following: 1. Catechin, 2. Epicatechin, 3. Paeoniflorin, 4. Calycosin, 5. Liquiritin, 6. Stilbeneglycoside, 7. Cinnamaldehyde, 8. Formononetin, 9. Glycyrrhizin. HPLC chromatograms of nine standards (I) and modified HGWD decoction (II) are shown. Chemical structures of assayed compounds in the modified HGWD are presented (III).
